# Sclerosing thymoma followed up for eight years as mediastinal goiter: A case report

**DOI:** 10.1016/j.ijscr.2020.02.034

**Published:** 2020-02-19

**Authors:** Yoshihito Iijima, Yuki Nakajima, Hiroyasu Kinoshita, Yasuyuki Kurihara, Yu Nishimura, Toshihiko Iizuka, Hirohiko Akiyama, Tomomi Hirata

**Affiliations:** a Division of Thoracic Surgery, Saitama Cancer Center, 780 Komuro, Ina-machi, Kita adachi-gun, Saitama 362-0806, Japan; b Division of Pathology, Saitama Cancer Center Japan, 780 Komuro, Ina-machi, Kita Adachi-gun, Saitama 362-0806, Japan

**Keywords:** ST, sclerosing thymoma, CT, computed tomography, Mediastinal tumor, Sclerosing thymoma, Hyalinization, Tumor regression, Thymus, Case report

## Abstract

**Introduction:**

Sclerosing thymoma (ST) is an extremely rare disease with less than 20 cases ever been described. Here, we present a case of sclerosing thymoma that was followed up as mediastinal goiter for eight years.

**Presentation of case:**

A 77-year-old man was presented with a superior mediastinal tumor. The patient was asymptomatic and not affected by myasthenia gravis. Computed tomography showed a well-defined superior mediastinal tumor whose size had regressed over time. Ultrasonography-guided core-needle biopsy revealed type B1 to B2 thymoma, and total-thymectomy was performed. Histopathologically, most of the tumor showed hyalinization and sclerosis, and slight signs of type AB thymoma were found at the tumor’s periphery. The patient was diagnosed with ST. No evidence of recurrence was observed 12 months following surgery.

**Discussion:**

Since sclerosing thymoma is mostly composed of fibrous tissue, small specimens such as needle biopsies do not contain tumor cell nests and are difficult to confirm. Complete resection is currently the most common treatment for ST. Spontaneous regression of ST has been reported; however, the mechanisms involved have not yet been elucidated.

**Conclusion:**

This rare case of sclerosing thymoma is an unusual case since it has follow up information for an eight year period due to the misdiagnosis of goiter. The follow up visits showed significant regression of the tumor over the eight year period without treatment; however, the etiology of sclerosis and regression remain unknown. The patient was treated by thymectomy with no recurrence after 12 months.

## Highlights

• We present an extremely rare variant of thymoma, known as sclerosing thymoma.
• Misdiagnosis as goitre provided follow up information for eight years.
• Regression of the tumor was significant prior to treatment with thymectomy.
• The patient recovered and is recurrence-free after 12 months.

## 1 Introduction

Thymoma originates within the epithelial cells of the thymus and is the most common anterior mediastinal neoplasm. The incidence of thymoma is 1.5 cases per million and comprises about half of the anterior mediastinal tumors. We present an extremely rare variant of thymoma, known as sclerosing thymoma (ST), which was first reported in 1994 by Kuo [1]. ST is defined as a thymoma that exhibits features of conventional thymoma, with abundant collagen-rich stroma [2]. ST is synonymous with “ancient thymoma" [3] and accounts for less than 1% of all thymomas; only 18 cases have been reported so far, including our case (16 cases in English and 2 cases in Japanese articles, See Table) [1,3,4]. Age of the patients ranged from 10 to 77 years (mean 50.1 years). Twelve subjects were males and six females. Myasthenia gravis was observed in four cases, three of whom were women [1,3,4]. Here, we present a case of ST that was followed up as mediastinal goiter for eight years in a 77-year-old man. The work has been reported in line with the SCARE criteria [5].

## 2 Presentation of case

**Fig. 1:**
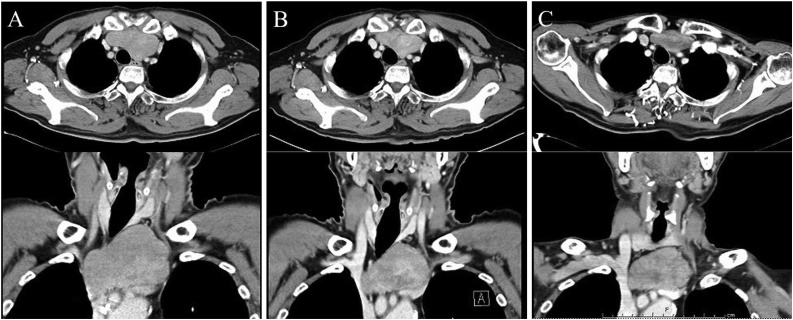
Change in tumor diameter on CT follow-up. The tumor size regressed from 7.3 × 4.3 × 5.2 cm according to the CT performed eight years ago (A) to 6.5 × 3.0 × 5.0 cm by CT carried out four years ago (B) and 5.7 × 2.7 × 4.0 cm in the latest CT taken before surgery (C).

**Fig. 2:**
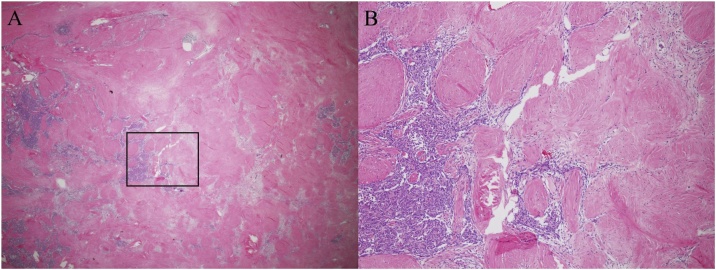
Microscopic findings. Histopathological examination showed type AB thymoma at the periphery of the tumor, which consisted of a component of type A thymoma with short spindle cells and oval cells and a component of type B1-2 thymoma with abundantly infiltrated immature T lymphocytes, magnification, ×2 (A) and magnification, ×10 (B).

A 77-year-old man was diagnosed with a superior mediastinal tumor while undergoing a tongue cancer examination and was referred to our division. He had a history of stomach cancer and myocardial infarction, and this mediastinal tumor had been followed up at the otorhinolaryngology department of another hospital for eight years as a mediastinal goiter. Although he was asymptomatic and had no myasthenia gravis, an elastic hard mass was observed on the dorsal side of the left clavicular head. Chest X-ray revealed that the trachea was slightly shifted to the right side. Laboratory tests for tumor markers, including carcinoembryonic antigen, squamous cell carcinoma antigen, carbohydrate antigen 19-9, alpha-fetoprotein, neuron-specific enolase, and soluble interleukin-2 receptor were all within normal ranges and the anti-acetylcholine receptor antibody was negative. Computed tomography (CT) of the chest revealed a well-circumscribed mass measuring 5.7 × 2.7 × 4.0 cm in the superior mediastinum that extended to the dorsal side of the left clavicular head (Fig. 1). A whole-body scan using 2-deoxy-2-(^18^F)-fluorodeoxyglucose-positron emission tomography/CT showed a maximum standardized uptake value of 2.87 by the mediastinal tumor. The tumor had regressed over the eight years, the original tumor size was 7.3 × 4.3 × 5.2 cm according to a CT taken eight years ago, 6.5 × 3.0 × 5.0 cm four years ago, with the current presentation at 5.7 × 2.7 × 4.0 cm (Fig. 1). An ultrasonography-guided core-needle biopsy was performed and revealed type B1 to B2 thymoma. Thus, it was diagnosed as clinically T1aN0M0 stage I (Masaoka stage I) thymoma and thymectomy through a median sternotomy was performed. The well-defined yellowish-white tumor measuring 60 × 55 × 30 mm was in the upper left pole of the thymus. Histological examination showed type AB thymoma mainly at the periphery of the tumor that consisted of a component of type A thymoma with short spindle cells and oval cells and a component of type B1-2 thymoma, which was abundantly infiltrated with immature T lymphocytes (Fig. 2). Although fibrosis was prominent in the peripheral area, there was a high degree of fibrosis accompanied by hyalinization in the central area and a region where the epithelial component had nearly disappeared. No hemorrhage or necrosis was detected. Immunohistochemistry results were as follows: epithelial cells were positive for AE1/AE3, weakly positive for Bcl-2, and negative for CD5 and c-kit; lymphocytes were positive for CD5, TdT, MIC2, and CD1a. This tumor exhibited rich collagenous fibrous stroma compared to a normal thymoma and was diagnosed as a ST; he showed no signs of recurrence 12 months following surgery.

## 3 Discussion

The tumor was dominated by a hyalinized, fibrosclerotic stroma that had expanded the septa, perivascular spaces, and tumor periphery; hence, specimen collection using techniques such as needle biopsy, typically does not yield a large volume of tumor cells, thus complicating the diagnosis. This case was diagnosed as thymoma by percutaneous needle biopsy, but the former doctor, who had also performed aspiration cytology, could not produce a conclusive diagnosis because tumor cells were not collected. Thus, the patient was initially diagnosed with goiter, which was followed-up for eight years. According to Kim et al., ST should be considered as a differential diagnosis when only fibrous tissue is collected during biopsies of the anterior mediastinal tumors [6]. However, Moran reported that 60% of patients who underwent a biopsy could not be diagnosed before their operation [3]. The biological behavior of ST remains mostly unknown. Follow-up information on reported cases showed no evidence of recurrence or metastasis and no tumor-related death. Complete resection is currently the most common treatment for ST.

While the etiology of ST was unclear, fibrogenic stimuli delivered by the neoplastic epithelium and/or regressive changes are thought to the elicit stromal expansion [2]. Based on histopathology analysis, Kuo et al. suggested that spontaneous regression of ST could occur [1]. Further, these reported tumors were present for a long time. According to Moran et al., during this period, the characteristic fibrocollagenous bands that are commonly observed in thymomas could have coalesced, with only the focal cellular areas remaining [3]. Although thymoma subtyping is often impossible, thymic areas having conventional type B2 and B3 thymoma are occasionally observed [2]. We summarized thymoma subtyping using the World Health Organization classification according to the descriptions provided in previously published studies. In these previous studies, three, two, one, seven, and two cases of type A, type AB, type B1, type B2, and type B3 thymomas have been reported (the other one case was not available), respectively (see Table 1). The incidence of type B2 thymoma was relatively high; however, all subtypes have been reported. In our case, slight signs of type AB thymoma were confirmed at the peripheral region of the tumor.

**Table 1:** Case reports sclerosing thymoma.

Case	Sex	Age (years)	Size (cm)	Clinical symptom	Myasthenia gravis	Thymoma subtype of WHO classification	Description of thymoma components and immunohistochemistry	Reference
1	F	39	3.0	Palpitation, Dyspnea	+	Type B3	epithelial type thymoma	[1]
2	F	23	2.5	muscle weakness, difficulty in talking	+	Type B1	lymphocytic type thymoma	
3	F	34	5.0	–	−	7 type B2 tumors and 3 type A tumors	7 tumors: the cellular aggregates composed of a dual cell population of epithelial cells and lymphocytes, no cellular atypia or mitotic activity. 3 tumors: the cellular aggregates were characterized by spindle cells with scant eosinophilic cytoplasm and absence of cellular atypia and mitotic activity.	[3]
4	F	62	8.0	–	−			
5	F	37	6.0	SOB, Chest pain	−			
6	F	27	5.0	–	+			
7	M	58	6.0	–	−			
8	M	44	5.0	–	−			
9	M	56	10.0	–	−			
10	M	69	7.0	SOB, Chest pain	−			
11	M	59	6.0	SOB, Chest pain	−			
12	M	73	10.0	SOB, Chest pain	−			
13	M	60	2.0	muscle weakness, difficulty in talking	+	Type A	medullary type, slightly lymphocytes infiltration, nomitotic activity, IHC: spindle cells; Keratin+, EMA+, Leu7+	[4]
14	M	47	2.0	–	−	Type AB	scattered, small aggregation of spindle to oval cells, mild lymphocytes infiltrate, no mitotic activity, IHC: AE1/AE3+	[5]
15	M	62	3.1	–	−	Type A	type A, IHC: spindle cells; AE1/AE3+, CD34-, lymphocytes; TdT+	[7]
16	M	10	7.0	Chest pain	−	N/A	–	[8]
17	M	65	4.9	–	−	Type B3?	IHC: Keratin+, p63+, Ki-67(20%), TdT-, CD1a-	
18	M	77	5.7	–	−	Type AB	IHC: epithelial cells; AE/AE3+, CD5-, c-kit-, bcl-2 weak+, lymphocytes; CD5+, TdT+, MIC2+, CD1a+	Our case

F: female, M: male, SOB: shortness of breath, N/A: not available, IHC: immunohistochemistry.

Various theories regarding the spontaneous regression of thymomas have been proposed. The mechanisms underlying this spontaneous regression included those related to the immune and endocrine systems, differentiation induction, removal of carcinogens, tumor necrosis, angiogenesis suppression, and apoptosis. Nonetheless, these mechanisms have not yet been completely elucidated [9,10]. Ito et al. reported a case of thymoma that spontaneously regressed, and the authors suggested that thymoma with hemorrhage and necrosis could transform into a sclerotic lesion as the necrosis component was absorbed over time to cause fibrosis [10]. In the current case, although there was no hemorrhage or necrosis in the tumor, fibrosis with extensive vitrification, and a few thymic tumor cells in the periphery were observed. It was unclear whether the terminal stage of this tumor had absorbed bleeding and necrosis, or whether another mechanism was involved. In contrast, there are no reports of the long-term observation of the natural progression of ST. Our case was followed up as mediastinal goiter for eight years, and the tumor regressed in size according to the CT follow-up, thereby supporting Kuo's suggestions [1]. Long-term degenerative changes might have resulted in tumor regression. The etiology of this tumor will be clarified in the future as more cases are evaluated.

## 4 Conclusion

We reported a case of ST. Although the tumor spontaneously regressed, the etiology of regression and sclerosis remain unknown. We confirmed the natural regression of this tumor over eight years by image analysis.

## Funding

All authors have no funding of research.

## Ethical approval

This study was approved by the institutional review board in June 2019 (approval number: 951), and the need to obtain informed consent was waived.

## Consent

Written informed consent was obtained from the patients for publication of this case report and accompanying images.

## Author contribution

Yoshihito Iijimaa carried out the operation, wrote this manuscript and carried out data collection. Yuki nakajima, Hiroyasu Kinoshita, Yasuyuki Kurihara, Yu Nishimura, Toshihiko Iizuka, Hirohiko Akiyama and Tomomi Hirata carried out the revision of the manuscript.

## Registration of research studies

N/A.

## Guarantor

Yoshihito Iijima.

## Availability of data statement

All of the data generated by this case is contained within the article.

## Provenance and peer review

Not commissioned, externally peer-reviewed.

## Declaration of Competing Interest

All authors report no conflict of interest.
